# Fibrous Stricture of the Rectum Relieved by Incisions and Elastic Pressure

**Published:** 1871-04

**Authors:** 


### Abridgments from our Exchanges.

FIBROUS STRICTURE OF THE RECTUM RELIEVED BY INCISIONS AND ELASTIC PRESSURE.—The following case is reported by Wm R. Whitehead, M.D., of New York, in the *American Journal of Medical Sciences:*

Mrs. P., æt. 38, was married nine years ago, and has been a widow since eight years; no children; one miscarriage at the fourth month; she has never been well since. A year after her marriage she noticed about the labia and anus some sores, which were long in healing, and which appeared successively one after the other. She frequently examined herself with a mirror. The sores finally healed, after persisting several months, and at one time she saw at the anus a crack, which was excessively painful, especially while at stool. Not long after the sores had healed, she was troubled with diarrhœa, and a muco-purulent discharge from the anus. During the last eighteen months this discharge was quite puriform, and at times escaped involuntarily, and compelled her to wear a napkin to prevent it from soiling her garments. Occasionally she had constipation with pain and flatulent distension of the abdomen, succeeded by diarrhœa and a more copious discharge of pus from the rectum. Her husband once had some trouble about his testes. Mrs. P. presented in her own history no evidences of constitutitional syphilis. There were no eruptions on the skin or about the throat. Defecation was usually difficult rather than painful, and the feces were flattened and about the thickness and width of a tapeworm.

The woman was pale, much emaciated, and had an exceedingly despondent look. There were hypertrophic masses of skin about the anus. The rectal touch revealed the presence of a very tight stricture just above the sphincter ani muscle, and about two inches from the anus. The first joint of the index-finger could only partially enter the stricture, causing great pain. As measured with the little finger, the stricture was conical, and about three-quarters of an inch in length. On different days several attempts at dilitation were ineffectually made, causing much pain. On the 20th of August the patient was etherized, placed in the "semi-prone" position, and the large blade of Sims' duck-bill speculum was hooked into the posterior segment of the sphincter ani muscle, which was forcibly retracted, and the speculum confided to an aid. The anterior part of the anus was depressed with the handle of a pair of scissors. This dilitation of the spincter ani muscle gave an excellent view of the stricture, which was tightly closed, and the shining fibrous borders of its lower orifice presented only a transverse linear depression. I cut the stricture, deeply and thoroughly, in the middle line, both posteriorly and in front, with a pair of stout, straight, and sharp-pointed scissors. The large blade of Sims' speculum, which measures 1⅝ of an inch in width, was really moved past the stricture. The hemorrhage which followed in the very free incisions was moderately profuse for a while, but soon ceased after some clots of blood were removed from the rectum and bits of ice applied to the bleeding surface. Every precaution was previously taken to avoid excessive hemorrhage, and the incisions were conducted with a due regard to the thickness of the recto-vaginal septum. The rectum was largely dilated so as to afford a good view of nearly the whole of its interior. This satisfactory exploration of the rectum was effected by the aid of Sims' speculum, which was held by my clinical assistant, Dr. Francis M. Deems, so as to very forcibly retract the posterior part of the sphincter muscle. A sponge about the size of a small orange, and secured to a string, was pushed up the rectum as high as the sigmoid flexure, and by distending the gut, aided the exploration, and at the same time afforded security against an undesirable "debacle" from above. The following suppository was left in the rectum: ℞ Pulv. opii. gr. iss; ext. belladon. gr. ⅛; olei theobromæ, q. s. Some brandy and a glass of milk-punch were given to the patient. She was left on the operating table a few hours, and afterwards walked home with the aid of her sister.

August 21.—Slept well during the night; she had one very large evacuation from her bowels, followed by several smaller ones. Ordered beef tea and milk-punch.

22d. 11 o'clock A.M.—She felt chilly early this morning; and slightly feverish; appetite impaired; diarrhœa. Passed No. 11 rectal bougie, then a bivalve speculum, the blades of which were separated 1¾ inch at their extremities. *This dilitation caused considerable pain and gave me some apprehension.* Tinct. cinch. co. ℥ss, ter die.

23d. 12.30 P.M.—Skin hot and dry; pulse 160 and small; no appetite; tongue dry, and great thirst; diarrhœa; complains of internal heat. ℞ Morphiæ sulphatis, gr. ij; spt. æther nitros. ℥ss; aquæ, ℥iss.—M. Teaspoonful every three hours. The rectum was syringed out with tepid water containing carbolic acid in the proportion of one drachm of acid to a quart of water. Within four hours after the first injection the pulse fell to 120; within ten minutes after the injection the patient felt much relieved. Beef-tea and milk-punch continued. Injections repeated.

24th. 11.30 A.M.—Skin moist, pulse 108; appetite improving. During the afternoon the pulse was 100. Condition very favorable. Carbolized injections in the rectum, washing it out thoroughly several times during the day.

25th.—Diarrhœa profuse. ℞ Pulv. Doveri, gr. xv; bismuth subnitratis ʒj; divided into six powders; one after each movement of the bowels. Milk-punch and beef-tea continued.

September 6.—She came to the dispensary and had a No. 12 rectal bougie passed into the rectum. The bougie was afterwards passed somewhat irregularly, and sometimes caused considerable pain, followed by fever.

I was much dissatisfied with the ordinary rectal bougies in use by surgeons, and was apprehensive lest I should perforate the soft and ulcerated mucous membrane above the stricture. This accident has occurred, and is liable to occur again, in the hands of even the most experienced surgeons who persist in using the rectal bougie.

I thought that possibly Barnes' dilators might be used for keeping the stricture dilated, but they were found to be too stiff, and unsuitable for the rectum. I knew that Bushe had used in the rectum a pig's bladder distended with water, for stopping hemorrhage from this gut after operations on it. I improvised an apparatus, which I have since much improved, and which consisted originally of what is commonly known as a "condom" or "capote" attached to the short pipe of Davidson's syringe, and provided with a stopcock. At first I used gut condoms, but have since substituted for them rubber capotes, which are better. They dilate the stricture without pain when slowly distended with warm water, and are tolerated by the rectum for hours without discomfort. If, however, the sphincter ani muscle is too much pressed on laterally by the lower part of the distended capote, there will be an effort to expel it. To obviate this occurrence, the pipe of the apparatus which is embraced by the sphincter should be at least one inch and a-quarter in length. Because there should be on the outside a smooth rubber flange to prevent the pipe from being drawn too far into the rectum.

October 10.—The health of Mrs. P. has improved in a most remarkable manner; she has large natural passages without pain or difficulty; she has gained much flesh, is quite cheerful, and profuse with her expressions of gratitude. The elastic dilitation is used about three times a week.

